# Designing Sustainable Packaging Materials: Citric Acid-Modified TPS/PLA Blends with Enhanced Functional and Eco-Performance

**DOI:** 10.3390/polym17192571

**Published:** 2025-09-23

**Authors:** Vesna Ocelić Bulatović, Mario Kovač, Dajana Kučić Grgić, Vilko Mandić, Antun Jozinović

**Affiliations:** 1 Faculty of Chemical Engineering and Technology, University of Zagreb, Trg Marka Marulića 19, 10000 Zagreb, Croatia; dkucic@fkit.unizg.hr (D.K.G.); vmandic@fkit.unizg.hr (V.M.); 2 Faculty of Agriculture and Food Technology, University of Mostar, Biskupa Čule bb, 88000 Mostar, Bosnia and Herzegovina; mario.kovac11@gmail.com; 3 Faculty of Food Technology Osijek, Josip Juraj Strossmayer University of Osijek, Franje Kuhača 18, 31000 Osijek, Croatia; ajozinovic@ptfos.hr

**Keywords:** potato starch, thermoplastic starch (TPS), polylactic acid (PLA), citric acid, biodegradable polymer blends, soil biodegradation, sustainable food packaging

## Abstract

Starch extracted from the domestically cultivated Scala potato variety was explored as a renewable resource for the formulation of biodegradable thermoplastic starch (TPS)/polylactic acid (PLA) blends intended for environmentally friendly food packaging applications. The isolated starch underwent comprehensive physicochemical and structural characterization to assess its suitability for polymer processing. TPS derived from Scala starch was compounded with PLA, both with and without citric acid (CA) as a green compatibilizer to enhance phase compatibility. The resulting polymer blends were systematically analyzed using Fourier-transform infrared spectroscopy with attenuated total reflectance (FTIR–ATR), scanning electron microscopy (SEM), differential scanning calorimetry (DSC), thermogravimetric analysis (TGA), and X-ray diffraction (XRD) to evaluate thermal and structural properties. Mechanical performance, water vapor permeability (WVP), water absorption (WA), and biodegradability in soil over 56 days were also assessed. The incorporation of citric acid improved phase miscibility, leading to enhanced structural uniformity, thermal stability, mechanical strength, and barrier efficiency. Bio-degradation tests confirmed the environmental compatibility of the developed blends. Overall, the results demonstrate the potential of Scala-based TPS/PLA systems, particularly those modified with citric acid, as viable candidates for sustainable food packaging, while highlighting the importance of further formulation optimization to balance functional and biodegradative performance.

## 1. Introduction

Growing global concern about environmental degradation and the continued accumulation of synthetic plastic waste has intensified the search for more sustainable and biodegradable materials, particularly in areas such as food packaging where single-use plastics dominate [1,2]. Conventional fossil fuel-derived plastics, while valued for their low cost, durability, and versatility, pose a significant environmental burden due to their degradability and the problems associated with their recycling [1,3,4,5]. In response to these pressing concerns, the development of biodegradable alternatives has become a major focus in materials science and offers a potential way forward that balances functional performance with environmental responsibility. Among the most promising candidates for sustainable packaging are materials derived from renewable biological sources, such as starch and polylactic acid (PLA). Starch, a naturally occurring carbohydrate found in many plants, can be combined with small molecules such as glycerol to create a thermoplastic material that is melt processable and moldable [5,6,7,8,9,10,11,12,13,14,15,16,17]. Thermoplastic starch (TPS) is widely regarded as an environmentally friendly material due to its renewability, low cost, and ability to decompose naturally in the environment [6,9,10,14]. However, its use is severely limited by its high affinity for water and low mechanical strength, making it unsuitable for many packaging applications [1,9,10]. Polymers derived from lactic acid, on the other hand, are also produced from renewable sources and are valued for their mechanical strength, optical clarity, and ease of processing [6,18]. Unlike starch-based materials, PLAs offer a more robust structure but also have their own drawbacks, including brittleness, slow degradation under natural environmental conditions, and relatively high production costs [5,6,18,19,20]. The idea of blending starch-based materials with PLAs stems from the desire to combine their complementary properties: the flexibility and biodegradability of starch with the strength and structural integrity of lactic acid-based polymers. However, this strategy poses a fundamental challenge, as the chemical and physical differences between these two types of polymers lead to poor compatibility. The lack of adhesion at the interface between the two materials leads to phase separation, weakening the mechanical properties of the blend and limiting its practical application [5,18,20]. To overcome these limitations, researchers have turned to the use of compatibilizers, substances that can promote better interaction between the different components of a polymer blend [5,6,18,19,20,21,22,23]. One particularly effective approach is the use of citric acid (CA), a naturally occurring organic acid known for its numerous reactive carboxyl and hydroxyl groups. When used in the production of starch-based materials, CA acts as both a crosslinking agent and a plasticizer [6,19]. Citric acid (CA), a naturally occurring organic acid with multiple reactive groups, is highly effective in modifying starch-based materials. Acting as both a crosslinker and plasticizer, CA forms ester and hydrogen bonds with starch, enhancing mechanical strength, thermal stability, and moisture resistance [19]. This modification also reduces plasticizer migration and increases elasticity. When blended with PLA, CA improves compatibility by strengthening interfacial adhesion, leading to better stress distribution, flexibility, and heat resistance. Additionally, CA promotes uniform dispersion and enhances processability [19]. These improvements also accelerate biodegradation, making CA-modified blends ideal for short-lived packaging applications [5,6,18,19,20,21,22,23]. This study presents a comprehensive investigation into the development and characterization of blends of citric acid-modified starch and lactic acid-based polymers. It examines how citric acid affects not only the individual properties of the starch but also the overall behavior of the blend, including mechanical performance, thermal behavior, moisture resistance, and rate of biodegradation. The results show that CA serves as a powerful natural compatibilizer and provides a practical and environmentally friendly means of overcoming the inherent incompatibilities between starch- and lactic acid-based materials. Although TPS/PLA blends and the use of citric acid have been studied previously, most reported works remain limited in scope, focusing either on isolated material properties or generic starch sources without integrating full-scale characterization and environmental assessment. In contrast, this study distinguishes itself by employing starch derived from the Scala potato variety, cultivated in Croatia’s fertile Slavonia region, and by conducting a comprehensive, multi-modal evaluation that links raw material origin, molecular interactions, functional performance, and biodegradability. This holistic approach has not yet been reported in the literature and therefore provides new insight into the optimization of bio-based blends for packaging purposes. Combining scientific knowledge and sustainable design, this study makes a significant contribution to the ongoing efforts to replace conventional plastics with biodegradable alternatives. It provides a deeper understanding of the molecular interactions that determine the performance of compounds, offers clues for optimizing material properties for industrial use, and highlights the potential of bio-based polymers as the basis for a new generation of environmentally friendly packaging solutions. By bridging raw material provenance with end-of-life biodegradability, this work establishes a stronger foundation for real-world applications in food packaging. Through innovative material design and rigorous assessment, it advances a future where functionality and sustainability align, working together to benefit both society and the environment.

## 2. Materials and Methods

### 2.1. Materials

Polylactic acid (PLA), specifically the Ingeo™ biopolymer 4043D, was used in this study, procured from NatureWorks LLC (Minnetonka, Minnesota, MN, USA). According to the manufacturer, it contains 4.3% of the D-isomer and has a melt flow rate (MFR) of 6.0 g 10 min^−1^ under standard conditions (210 °C with a load of 2.16 kg). The polymer has a 1.24 g cm^−3^ density, an average molecular weight (*M_w_*) of approximately 168.000 g mol^−1^, a melting temperature between 145 and 160 °C, and a glass transition temperature between 55 and 60 °C. The native potato starch used in this study was isolated from a potato cultivar with the code name Scala. Details of the extraction method and physicochemical characterization of this starch can be found in our previous publication [24]. Table 1, provided below, summarizes the key physicochemical parameters of the Scala variety, as previously reported in our earlier study [24]. Glycerol with a purity of 99.8% (analytical grade) was purchased from Gram-Mol (Zagreb, Croatia) and served as a plasticizer for the native starch. The citric acid monohydrate (analytical grade) used in the experiments was also supplied by Gram-Mol (Zagreb, Croatia).

### 2.2. Thermoplasticization of Native Starch and Structural Modification via Citric Acid

Thermoplastic starch (TPS) was produced by blending native potato starch (from the Scala cultivar) with glycerol as a plasticizer in a 60/40 ratios (starch to glycerol). The blends were processed using a laboratory-scale single-screw extruder (Model 19/20DN; Brabender GmbH, Duisburg, Germany) with a 4 mm round die. The extruder barrel temperatures were maintained at 100 °C in the dosing zone, 100 °C in the compression zone, and 130 °C in the discharge zone. After extrusion, the material was cut into small pieces and granulated into pellets. Potato starch citrates were synthesized following the methodology described by Kapelko-Zeberska et al. [25], incorporating citric acid at concentrations of 5, 10, and 20 wt.%. The modified thermoplastic starch, referred to as mTPS_XCA (where X denotes the citric acid content), was prepared using the same extrusion process as TPS.

### 2.3. Fabrication of PLA-Based Binary Blends

After the preparation of thermoplastic starch (TPS) and its form modified with citric acid (mTPS_XCA), binary blends with PLA were prepared in a two-step process. First, the thermoplastic starch was dried at 105 °C for 24 h to remove the residual moisture content. In the first stage of the blending process, different proportions of TPS or mTPS_XCA (40, 50, and 60 wt.%) were manually blended with PLA pellets in polyethylene bags for about 5 min to obtain a preliminary blend (Table 2). The blends were then transferred to a Brabender kneader (Brabender GmbH & Co. KG, Duisburg, Germany) preheated to 170 °C. Blending was carried out at a rotor speed of 60 revolutions per minute (rpm) for 5 min to ensure homogeneous dispersion of the components. The resulting mixtures were then molded using a hydraulic laboratory press (Model SRB 140, EC 320 × 320 NB, Fontijne Presses, Rotterdam, The Netherlands) at 180 °C and a pressure of 25 kPa. The pressing cycle consisted of a 1-min preheating phase, a 5-min compression phase and a 20-min cooling phase at constant pressure. Pure PLA, TPS, and mTPS_XCA were also subjected to the same thermal and mechanical processing conditions and served as reference materials. The dimensions of the molds were selected according to the specific test requirements. For the mechanical, thermal, and structural analyses, the blends were molded into rectangular test specimens with dimensions of 10 mm × 10 mm × 1 mm. In contrast, the materials for the soil biodegradation experiments and the subsequent morphological and spectroscopic investigations were formed into thin films with a thickness of approximately 0.4 mm by compressing the sample granules between two plates on a laboratory-scale hydraulic press. The film thickness varied depending on the starch content, with higher TPS values resulting in thicker films. These films were then cut into square samples (15 mm × 15 mm) and weighed using a high-precision analytical balance (SAB 125i, Adam Equipment Co., Ltd., Milton Keynes, UK) with an accuracy of 0.00001 g.

### 2.4. FTIR–ATR Spectroscopy

The FTIR–ATR analysis of the TPS/PLA blends was performed over a range of wavenumbers 4000–650 cm^−1^ on a PerkinElmer (Waltham, MA, USA) Spectrum One FT-IR spectrometer using an attenuated total reflectance (ATR) accessory at a resolution of 4 cm^−1^ by four scans.

### 2.5. Melt Flow Index (MFI)

The melt flow index (MFI) of pure polymers and blends was measured using a Zwick 4100 capillary rheometer (ZwickRoell GmbH & Co., KG, Ulm, Germany) equipped with a capillary die of 8 mm in length and 2 mm in diameter. For each measurement, 7 g of the sample was placed into the instrument’s cylinder and preheated for 6 min before being heated to the test temperature of 190 °C. A force was applied to the piston using a 2.16 kg weight, extruding the molten sample through the capillary die. The extrudates were collected and weighed, and the MFI values were expressed in g 10 min^−1^. Each sample was tested three times to ensure repeatability. For TPS and mTPS samples, the same procedure was followed, but the measurements were conducted at a temperature of 160 °C.

### 2.6. Morphology

The morphological characteristics of the blends were analyzed using a Vega 3 scanning electron microscope (TESCAN, Brno, Czech Republic). Cross-sections of cryogenically fractured surfaces of the molded bars were examined to assess their microstructure. Before imaging, the samples were immersed in liquid nitrogen and subsequently fractured to obtain clean fracture surfaces. To prevent charging effects under the electron beam, the specimens were coated with a thin layer of gold. SEM analysis was conducted at an accelerating voltage of 20 kV.

### 2.7. Thermal Stability

#### 2.7.1. TGA Analysis

The thermal stability of films was determined by thermogravimetric analysis (TGA) using a TGA Q500 (TA Instruments, New Castle, DE, USA). The samples of approximately 10 mg were heated from 25 °C to 600 °C at a heating rate of 10 °C min^−1^ under a nitrogen purge at a flow rate of 60 mL min^−1^. Weight loss was shown as a function of temperature.

#### 2.7.2. DSC Analysis

The thermal properties of the blends were analyzed using a DSC 823e differential scanning calorimeter (Mettler Toledo, Greifensee, Switzerland) equipped with an intercooler cooling system. Approximately 10 mg of each sample was placed in hermetically sealed aluminium pans to prevent moisture loss. Throughout the analysis, a nitrogen purge flow of 50 mL min^−1^ was maintained. The samples were initially heated from room temperature to 200 °C at a heating rate of 10 °C min^−1^, held at 200 °C for 5 min for isothermal stabilization, and then cooled to -90 °C at the same rate. After equilibration at -90 °C, the samples were reheated to 200 °C at 10 °C min^−1^. Crystallization and melting parameters were extracted from the second cooling and reheating scans. The degree of crystallinity (*χ_c_*) of the blends was calculated from the melting enthalpy of the crystalline phase of PLA in the secondary heating curves (Δ*H_m_*) according to Equation (1):

(1) $$\chi_{c}\left(\%\right)=\left(\Delta H_{m}\right)/\left(∆H_{m}^{0}\times w\left(PLA\right)\right)\times 100$$

where *w*(PLA) represents the mass fraction of PLA (%), Δ*H_m_* denotes the melting enthalpy of the crystalline phase of PLA (J·g^−1^), and Δ*H_m_^0^* corresponds to the melting enthalpy of fully crystalline PLA (J·g^−1^), with a reference value of 93.1 J·g^−1^ [26].

### 2.8. X-Ray Diffraction Analysis

The blends of thermoplastic native starch of the Scala variety and thereof prepared starch citrates (mTPS) with polylactic acid (PLA) were analyzed by X-ray diffraction analysis using diffractometer D8 Advance (Bruker, Billerica, MA, USA) operated in Bragg–Brentano configuration at accelerating voltage of 40 kV and current 25 mA, between 10 and 80°2θ with a scan speed of 0.6°2θ min^−1^.

### 2.9. Water Absorption

Water absorption (*W_a_*) was determined using rectangular specimens with dimensions of 20 × 10 × 1 mm^3^, obtained during sample preparation via hydraulic pressing (Model SRB 140, EC 320 × 320 NB, Fontijne Presses, Rotterdam, The Netherlands) for characterization purposes. Before measurement, the specimens were dried overnight in a vacuum oven at 50 °C. After drying, they were immediately weighed (*m_0_*/g) and immersed in distilled water at room temperature (~23 °C) with 100% relative humidity. Over six days, the specimens were removed from the water every 24 h, gently blotted with a paper towel to remove excess surface water, and subsequently weighed (*m_t_*/g) to determine water absorption (*W_a_*/%) using Equation (2). However, for TPS and mTPS samples, due to their rapid water absorption and structural disintegration, the measurement period was limited to 10 h instead of six days.

(2) $$W_{a}\left(\%\right)=((m_{t}-m_{0})/m_{0})\times 100$$

### 2.10. Water Vapor Permeability (WVP) According to Herfeld

The water vapor permeability (WVP) of pure TPS, PLA, and their biodegradable polymer blends was determined using a Herfeld apparatus, following the method described by Bota et al. [27]. The device consisted of a glass container with a metal lid featuring a circular opening with a diameter of 36 mm. A volume of 50 cm^3^ of distilled water was poured into the container, and a circular thin film of the test sample (55 mm in diameter) was placed on the lid with its functional (upper) side facing upward. Once the lid was secured, the apparatus was placed inside a desiccator containing 97% sulfuric acid (H_2_SO_4_). The mass of the apparatus, including the test film and water, was measured at the beginning of the test, as well as after 24 and 48 h, to determine the water vapor permeability. The WVP value is calculated using Equation (3).

(3) $$PVP\;\left(g\cdot m^{-2}\cdot {dan}^{-1}\right)=((m_{0}-(m_{1}+m_{2})/2)/A$$

where *m_0_* (g) denotes the mass of the apparatus containing water and the test tube at the beginning of the measurement. The masses recorded after 24 and 48 h are represented by *m_1_* (g) and *m_2_* (g), respectively. The exposed surface area (*A*) of the test film is calculated using the equation *A* (m^2^) = *r*^2^π, where *r* (m) is the radius of the circular film sample.

### 2.11. Mechanical Properties

The tensile properties of the blends were evaluated using a Zwick 147670 Z100/SN5A universal testing machine (ZwickRoell GmbH & Co., KG, Ulm, Germany) equipped with a 2000 N load cell. The tests were conducted at an ambient temperature of 23 °C, following the ISO 527-1:2019 standard [28]. All specimens were conditioned for 72 h at 23 °C and 50% relative humidity before testing. A crosshead speed of 50 mm min^−1^ was applied to determine tensile strength, elongation at break, and elastic modulus. The thickness of each sample was measured at three different points, and the average value was used for calculations. For statistical reliability, a minimum of five specimens per sample were tested, and the reported results represent mean values with a standard deviation of less than 5%.

### 2.12. Biodegradability Analysis of the Material

The biodegradability assessment was conducted following the ISO 17556:2019 standard [29]. Samples were placed in reactors containing 300 g of moist soil with 60% humidity. Each sample was positioned in a plastic container, covered with an additional 300 g of moist soil, and sealed with a protective film to maintain soil moisture, as previously described and illustrated in reference [30]. This approach ensured that the experiment was carried out under controlled conditions following international standards, allowing for detailed monitoring of the material’s biodegradation process. For easier identification, each sample was labeled before being removed from its container. This labeling ensured accurate tracking of each specimen throughout the entire process, contributing to the precision of the analysis and consistency of the results. Before testing, the soil was enriched with a mixed microbial consortium, including *Pseudomonas aeruginosa*, *Bacillus* sp., *Trichoderma* sp., *Aspergillus niger*, and *Saccharomyces cerevisiae*. The microbial suspension (50 cm^3^ total volume) was prepared from environmental samples maintained in the Collection of the Department of Industrial Ecology at the University of Zagreb Faculty of Chemical Engineering and Technology. Polymer blend samples were incubated in a thermostat at 58 °C, and the soil was regularly moistened with deionized water. Samples were retrieved from the soil at predetermined time intervals, on days 7, 14, 21, 28, 42, and 56. Upon removal, the samples were initially subjected to microscopic examination was performed using an Olympus BX50 light microscope equipped with an Olympus DP10 camera (Olympus Corporation, Tokyo, Japan). This analysis was conducted before the washing step with 70% ethanol and deionized water and enabled detailed observation of the surface morphology and structural changes in the samples before further processing. After the initial microscopic assessment, the samples were thoroughly washed with 70% ethanol (p.a.) and deionized water to remove residual organic matter and soil particles, then air-dried for 120 min before weighing. Throughout the biodegradation process, the mass loss of the polymer blends was monitored and calculated using a specific mathematical equation. Following the biodegradation period, the samples were further analyzed using polarized light microscopy to assess internal morphological changes and possible crystalline structure alterations. This examination was performed using an Olympus BX53M microscope equipped with polarization optics and an Olympus SC50 camera (Olympus Corporation, Tokyo, Japan). Observations were conducted at a total magnification of 100×, allowing for a detailed evaluation of the polymer blends’ microstructural features after degradation. Throughout the biodegradation process, the mass change (Δ*m*/%) of the polymer blends was systematically monitored and quantified using a corresponding mathematical expression to evaluate the extent of degradation over time, Equation (4).

(4) $$∆m\;\left(\%\right)=({m\_}_{before}-{m\_}_{after})/{m\_}_{before}\times 100$$

where Δ*m* denote as percentage change in mass loss (%), *m__before_* is initial mass of the sample before biodegradation (g), and *m__after_* is mass of the sample after biodegradation (g).

## 3. Results

### 3.1. FTIR–ATR Analysis of TPS and TPS/PLA Biopolymeric Blends

**Figure 1:**
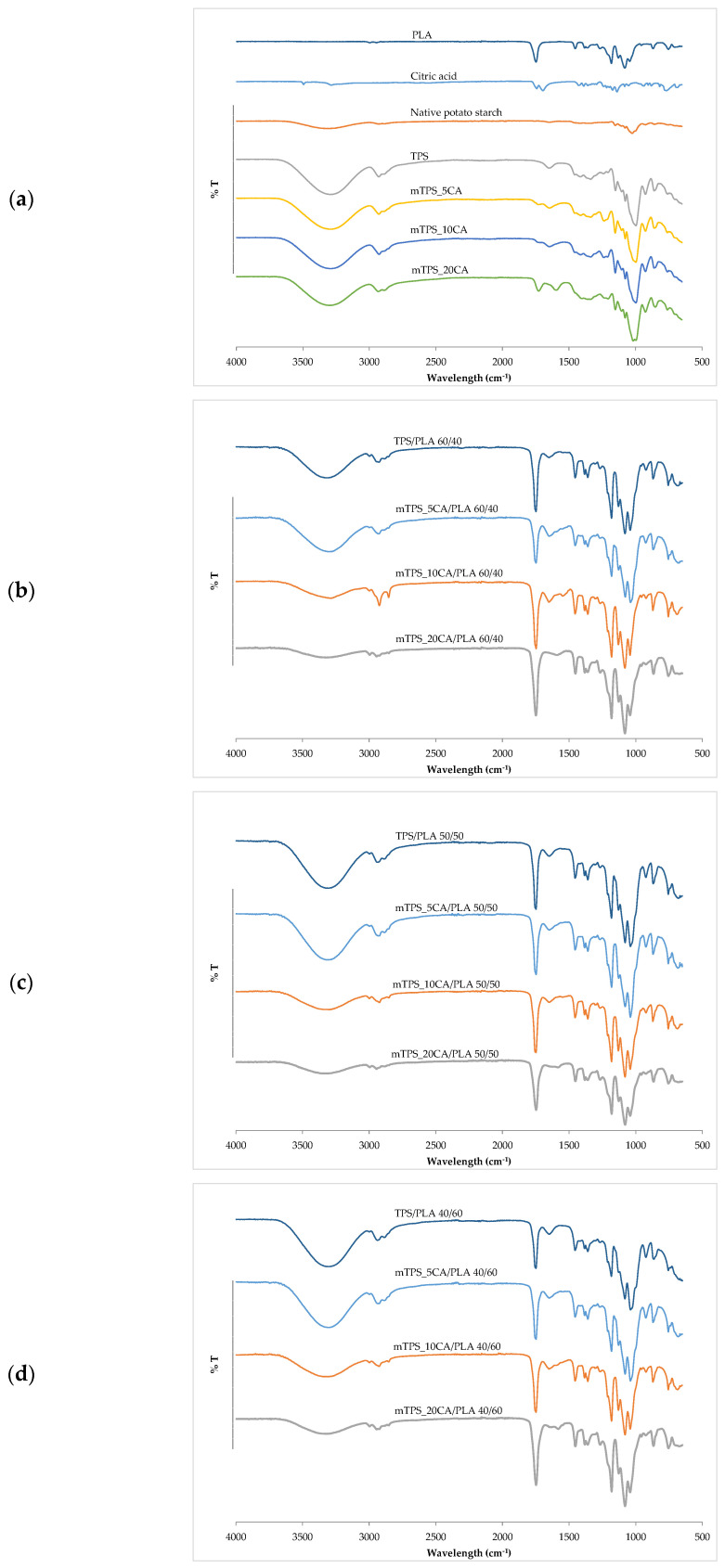
FTIR spectra of (**a**) PLA, citric acid, native potato starch, TPS, and mTPS; (**b**) TPS/PLA and mTPS/PLA 60/40; (**c**) TPS/PLA and mTPS/PLA 50/50; and (**d**) TPS/PLA and mTPS/PLA 40/60 biopolymeric blends.

FTIR–ATR spectroscopy was used to analyze the chemical structure and confirm esterification between thermoplastic starch and citric acid. This technique identifies functional groups and reveals structural changes key to optimizing TPS for sustainable packaging. The esterification was confirmed by comparing the infrared spectra of mTPS_XCA with spectra of unmodified starch, citric acid, and reference data from the literature [31,32,33]. Characteristic spectral features of ester bonds as well as shifts and enhancements of key bands serve as indicators of successful chemical modification (Figure 1). In the FTIR–ATR spectra of PLA, a biopolyester used as a blending matrix, clearly defined ester-related bands can be observed. The strong absorption band at 1746 cm^−1^ corresponds to the stretching vibration of the ester carbonyl group (C=O). Further bands at 2998 and 2852 cm^−1^ represent the symmetric and asymmetric C–H stretching of methyl groups, while deformation vibrations of –CH_3_ can be seen at 1390 and 1360 cm^−1^. A broad –OH stretching band at 3300 cm^−1^, attributed to hydrogen bonding, is seen in both PLA and mTPS samples and reflects the hydrophilic character and intermolecular interactions, particularly between hydroxyl groups and plasticizers, such as glycerol, and with citrate esters formed during esterification. In the case of mTPS, the FTIR–ATR spectra show clear evidence of ester formation upon the addition of citric acid [32]. In mTPS_5CA, new absorption maxima are observed at 1730 cm^−1^ (C=O stretching of the ester) and at 1234 and 1211 cm^−1^ (C–O stretching of the ester groups). These features confirm the occurrence of esterification. Similar trends are observed for mTPS_10CA, where the corresponding ester bands are at 1724, 1238, and 1207 cm^−1^, again confirming the formation of covalent bonds between the hydroxyl groups of the starch and the citric acid moieties. These observations are consistent with the well-documented reaction pathway in which citric acid dehydrates under heating to form citric anhydride, which preferentially reacts with the primary hydroxyl groups (especially at C–6) of the starch glucose moieties and forms ester bonds [34,35]. The spectra of TPS/PLA blends confirm the structural integration of PLA and TPS. Blends with different compositions (60/40, 50/50, and 40/60 TPS/PLA ratios) show characteristic peaks of both components. In all mixture formulations, the presence of esterification indicators is maintained, and differences in peak intensity reflect different levels of citric acid and starch. For example, in the mTPS_10CA/PLA 60/40 blend, an enhanced peak at 2924 cm^−1^ indicates increased C–H bond stretching in the TPS chain [36]. Similarly, the FTIR–ATR spectra of mTPS_10CA/PLA 50/50 and 40/60 show an enhancement of both PLA- and TPS-related bands. In particular, in the mTPS_10CA/PLA 40/60 blend, an increase in absorbance at 1748 cm^−1^ indicates increased C=O stretching of the PLA matrix. In addition, the peaks at 1181, 1129, and 1080 cm^−1^, which are assigned to C–O bond stretching [20], are more pronounced, indicating increased interaction and compatibility between PLA and esterified mTPS. Overall, the FTIR–ATR analysis confirms that CA effectively induces the esterification of starch, changing the chemical structure and allowing better interaction with PLA in blend systems. These molecular changes are critical for improving the performance characteristics of biodegradable polymer blends intended for sustainable applications.

### 3.2. Melt Flow Index (MFI) and Rheological Behavior of TPS/PLA Blends

The melt flow index (MFI) is a critical rheological parameter that reflects the flowability of thermoplastic materials under standardized conditions. It is inversely related to the viscosity and molecular weight of the polymer system and is often used in industry to assess processability and ensure consistent polymer quality before production. Table 3 shows the MFI results for thermoplastic starch derived from the Scala potato variety and its polymer blends with PLA. As expected, pure PLA showed the highest MFI value (6.3 g (10 min)^−1^), indicating a lower viscosity and better flow properties due to its aliphatic polyester structure. For the TPS/PLA blends, a constant increase in MFI was observed with increasing PLA content. This trend reflects the incorporation of the less viscous PLA phase into the more viscous TPS matrix, which improves the overall flow behavior [37,38]. The addition of citric acid, which is used as a plasticizer and esterifying agent, also influenced the rheological properties of the blends. Compared to pure TPS, all formulations with PLA and citric acid showed an increased MFI, indicating lower viscosity and better processability. The addition of citric acid led to more pronounced and non-linear changes in the MFI for the Scala variety. In TPS/PLA 60/40 and 50/50 blends, increasing the citric acid content from 5 to 10 wt.% led to a decrease in the MFI. However, a further increase in the citric acid content to 20 wt.% caused the MFI to rise again. In contrast, mTPS_XCA/PLA 40/60 blends showed only a slight, gradual increase in MFI with increasing citric acid content. These differences indicate that the interaction between citric acid and starch depends on the composition and is influenced by the specific structural properties of the starch. It is known that citric acid esterifies hydroxyl groups in starch and simultaneously acts as a mild plasticizer and compatibilizer. This dual role can alter the intermolecular interactions, reduce the interfacial tension between the polar TPS and non-polar PLA phases, and change the viscosity of the blend [38,39]. The transient decrease in MFI observed at intermediate citric acid concentrations (10 wt.%) could indicate increased hydrogen bonding or partial network formation, which temporarily increases the viscosity of the blend. At higher concentrations (20 wt.%), the softening effect of citric acid probably dominates, reducing intermolecular interactions and promoting chain mobility, which increases the MFI again. These observations underline the importance of the botanical origin of the starch for the rheological behavior [40]. The structure and molecular weight distribution of Scala starch appear to interact more dynamically with citric acid compared to other varieties, highlighting the importance of variety selection in tailoring the flow and processing properties of TPS-based materials. The Scala variety shows a pronounced rheological response to PLA blending and citric acid modification. The observed changes in melt flow index reflect a combination of compositional effects, interactions between starch and citric acid, and the inherent structural properties of Scala-based TPS. These results support the use of Scala starch as a viable and tunable matrix for biodegradable polymer blends, particularly when processing performance is to be optimized through controlled plasticization and compatibilization strategies.

### 3.3. Morphological Analysis of TPS/PLA Biopolymeric Blends Using Scanning Electron Microscopy

**Figure 2:**
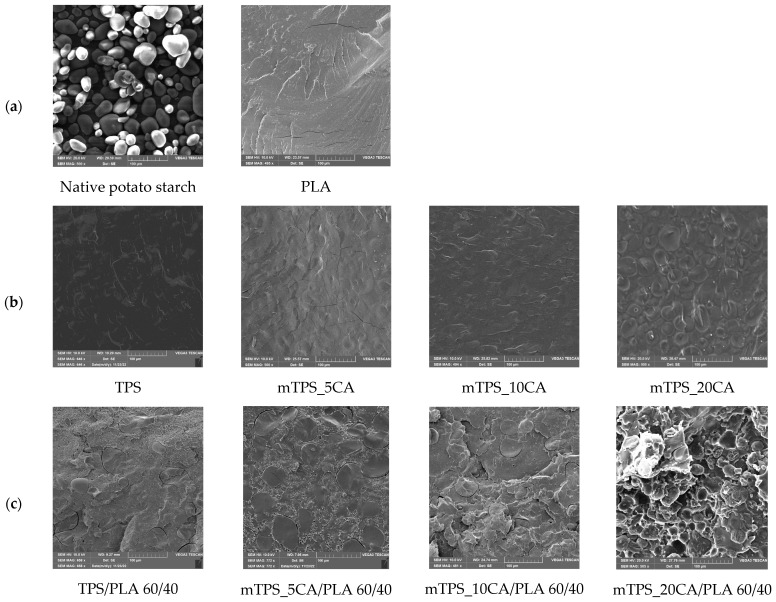

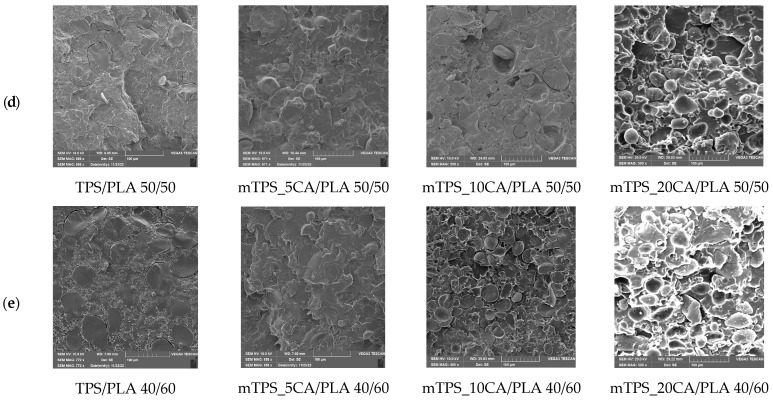
SEM micrographs of (**a**) native potato starch and PLA; (**b**) TPS and mTPS; (**c**) TPS/PLA and mTPS/PLA 60/40; (**d**) TPS/PLA and mTPS/PLA 50/50; and (**e**) TPS/PLA and mTPS/PLA 40/60 biopolymeric blends.

SEM was used to study the morphology of TPS and its blends with PLA, both with and without the addition of citric acid. The fracture surfaces of the polymer samples obtained by cryogenic fracturing in liquid nitrogen were analyzed to evaluate the compatibility and interfacial adhesion between TPS and PLA (Figure 2). SEM micrographs of TPS/PLA 40/60 (Figure 2) showed a pronounced biphasic morphology, indicating poor compatibility between the hydrophilic TPS and the hydrophobic PLA. The TPS granules appeared clearly separated in the PLA matrix, indicating limited interfacial adhesion. The addition of 5 wt.% CA slightly improved the morphological uniformity by reducing the phase separation of the starch, resulting in a more homogeneous structure, although the phase boundaries remained visible. However, at higher CA concentrations (10 and 20 wt.%), strong extrusion and agglomeration of the starch granules was observed. This suggests that increasing the citric acid content beyond 5 wt.% did not improve interfacial adhesion and may even have disturbed the polymer matrix, possibly affecting mechanical performance. Figure 2d shows the SEM micrographs of TPS/PLA 50/50 blends. Without CA, clear phase boundaries were again observed. After adding 5 wt.% CA, the morphology became more continuous, with less pronounced phase separation, suggesting improved interfacial interaction. This blend showed the best morphological compatibility among all formulations tested, with less pore formation and improved dispersion of the TPS domains. However, at 10 wt.% CA, the starch granules were increasingly expelled from the PLA matrix, leading to the appearance of voids, potential nucleation sites for moisture entrapment and microbial growth. This confirms that too much CA compromises the structural integrity of the blend by weakening adhesion, as was also found in previous studies [40,41]. SEM micrographs of TPS/PLA 60/40 blends (Figure 2c) revealed TPS-rich structures with large, unevenly distributed starch domains. These domains exhibited a rough morphology and well-defined phase boundaries, indicating limited miscibility and weak adhesion between TPS and PLA. The micrograph of the mixture with 5 wt.% CA showed a slightly improved dispersion but still a discontinuous structure with different TPS domain sizes. By increasing the CA content to 10 wt.%, the phase boundaries and heterogeneity of the TPS dispersion became even clearer. These results confirm that a high TPS content reduces compatibility and that the addition of CA alone cannot completely overcome the inherent polarity mismatch between TPS and PLA at this ratio. The SEM analysis consistently showed that the morphological compatibility between TPS and PLA is composition and plasticizer dependent. While all TPS/PLA blends exhibited clear phase separation due to polarity differences, the addition of 5 wt.% citric acid resulted in the clearest improvements in interfacial adhesion and morphological homogeneity, particularly at the mTPS_5CA/PLA 50/50 blend ratio. Higher concentrations of CA (10 and 20 wt.%) generally resulted in poorer morphology characterized by agglomeration of starch granules, voids, and lower miscibility. These results suggest that 5 wt.% CA is optimal for improving the compatibility of TPS/PLA blends, particularly at balanced blending ratios. The effects align with citric acid’s dual role as a mild plasticizer and compatibilizer, promoting esterification with starch hydroxyl groups and modifying interfacial tension. However, excessive plasticization can undermine matrix cohesion and phase distribution, underscoring the need to tailor additive levels to starch type and blend composition.

### 3.4. XRD Analysis of the Biopolymeric Blends

**Figure 3:**
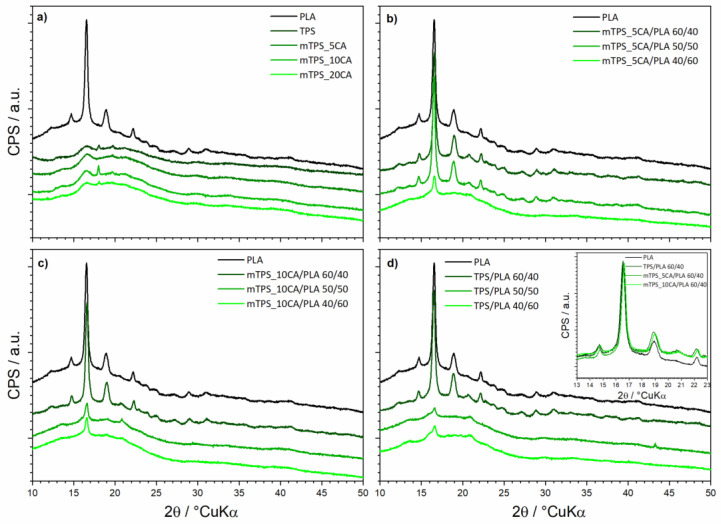
XRD diffraction of all constituents and biopolymeric blends: (**a**) PLA, TPS and differently esterified TPS; (**b**) PLA and different blends of 5%-esterified-TPS/PLA; (**c**) PLA and different blends of 10%-esterified-TPS/PLA; (**d**) PLA and different blends of unesterified-TPS/PLA; Inset: Comparison of PLA and TPS/PLA = 60/40 blends.

PLA is crystalline, and it can be easily observable in the difractograms via major peaks at 14.7, 16.6, 19.0, 20.7, and 22.2° 2θ (Figure 3a). When blended with TPS, the TPS can nucleate further growth of PLA and even increase its crystallinity [30]. In addition to used TPS and PLA in different ratios (40/60, 50/50, and 60/40), the system contained glycerol (40% w V^−1^) as a plasticizer. Reasonably enough, the crystalline residuals of glycerol were not found in the analyzed samples. However, the plasticizers can affect the structure of the TPS, usually in a disruptive manner [42,43]. This did not happen, so we can conclude the optimal amount of plasticizer was used. TPS is in principle a semicrystalline biopolymer. In our case, the TPS is clearly semicrystalline (Figure 3a); in addition to the amorphous hump centered at about 20° 2θ, there are only a few observable peaks centered at 13.5, 16.5, 18.0, and 19.5° 2θ. The esterification process (by addition of CA) should diminish the crystallinity of a polymer; replacing hydroxyl groups (–OH) on the starch molecules with ester groups (–COOR) usually disrupts the ordered structure of native starch. The purpose of the esterification is to increase plasticity and decrease hydrophilicity of TSP [30]. In principle, maintaining some crystallinity of TPS should allow for better tensile strength and stiffness properties, as well as degradation resistance and general stability, while still allowing for good workability and water resistance. Our system contained different amounts of citric acid used to esterify the native TPS (samples mTPS_5CA, mTPS_10CA, mTPS_20CA). The crystalline residuals and derivatives of the citric phase were not found in the system. Samples mTPS_5CA and mTPS_20CA prove the disruption of the structure; however, that is not the case for sample mTPS_10CA, which interestingly points to the increase of structural ordering of TPS (Figure 1). There are two possible explanations. A much more surface-contained esterification for the sample mTPS_10CA would explain maintaining the initial structural order [44]. The relevant surface performance would be unfazed, while bulk properties, such as the crystallinity of the TPS, would be hardly affected, as recorded by a surface-insensitive XRD analysis. However, the actual increase in the crystallinity of TPS can be explained only by a new formation of ester groups at higher substitution levels. Despite being much rarer, the increase in the TPS crystallinity has been evidenced in the literature and has been attributed to either very gradual esterification or to presence of specific ester groups or/and additives, ultimately causing the increase in chain entanglement and consequently promoting crystallinity [45]. Our results suggest that initial esterification heavily disrupts the initial crystalline structure of starch (mTPS_5CA); thereafter, the higher presence of ester groups facilitates structural ordering (mTPS_10CA). Finally, for the highest concentration of ester groups (mTPS_20CA), the disruptive effect is again visible. The crystallinity of the mTPS_20CA sample is still somewhat higher than that of the mTPS_5CA, serving as further evidence for the inflection hypothesis. Following these observations, only the blends with native TPS and TPS esterified with 0.05 and 0.10 g g^−1^ of citrate were further analyzed. Good blending quality generally leads to an increase in the crystallinity of all phases of the blends, where proper mixing and compatibilization reflects better ordering of the constituting chains, i.e., better structural order [46]. TPS can even nucleate additional growth of the PLA [30]. On the contrary, phase separation will be accompanied by lowered crystallinity and diminishing of the desirable properties of the composite. In all blends, just the PLA is differently crystalline, while TPS is fully amorphous. As previously said, TPS and PLA were blended in different ratios: 40/60, 50/50, and 60/40, samples TPS/PLA 40/60, TPS/PLA 50/50, and TPS/PLA 60/40. Blends TPS/PLA 40/60 and TPS/PLA 50/50 showed considerable phase separation evidenced in strong reduction of the crystallinity of PLA. This is also evidenced though change of the color and lowered transparency of the composite stripes. However, the blend TPS/PLA 60/40 improved the crystallinity of PLA beyond the initial PLA, pointing to great blending conditions (Figure 3b). For biopolymeric blends of PLA and mTPS (samples mTPS_5CA/PLA 40/60, mTPS_5CA/PLA 50/50, mTPS_5CA/PLA 60/40), the behavior was somewhat different. Only the blend mTPS_5CA/PLA 40/60 showed the phase separation, while both blends TPS_5CA/PLA 50/50 and mTPS_5CA/PLA 60/40 improved crystallinity of PLA (again beyond the initial PLA), the blend mTPS_5CA/PLA 60/40 marginally better (Figure 3c). For blends of PLA and mTPS (samples mTPS_10CA/PLA 40/60, mTPS_10CA/PLA 50/50, and mTPS_10CA/PLA 60/40), which was initially the mTPS version with the highest crystallinity, the behavior followed the same principle. The blends with mTPS_10CA/PLA 40/60 and mTPS_10CA/PLA 50/50 showed strong and moderate phase separation, respectively, while the blend with mTPS_10CA/PLA 60/40 heavily improved crystallinity (Figure 1). Esterifying TPS hardly has any effect on promoting the crystallinity of PLA in blends. On the other hand, having at least 60% of mTPS seems to be necessary to promote the crystallinity of PLA in biopolymeric blends (Figure 3d Inset).

### 3.5. Thermal Behavior of TPS/PLA Biopolymeric Blends

**Figure 4:**
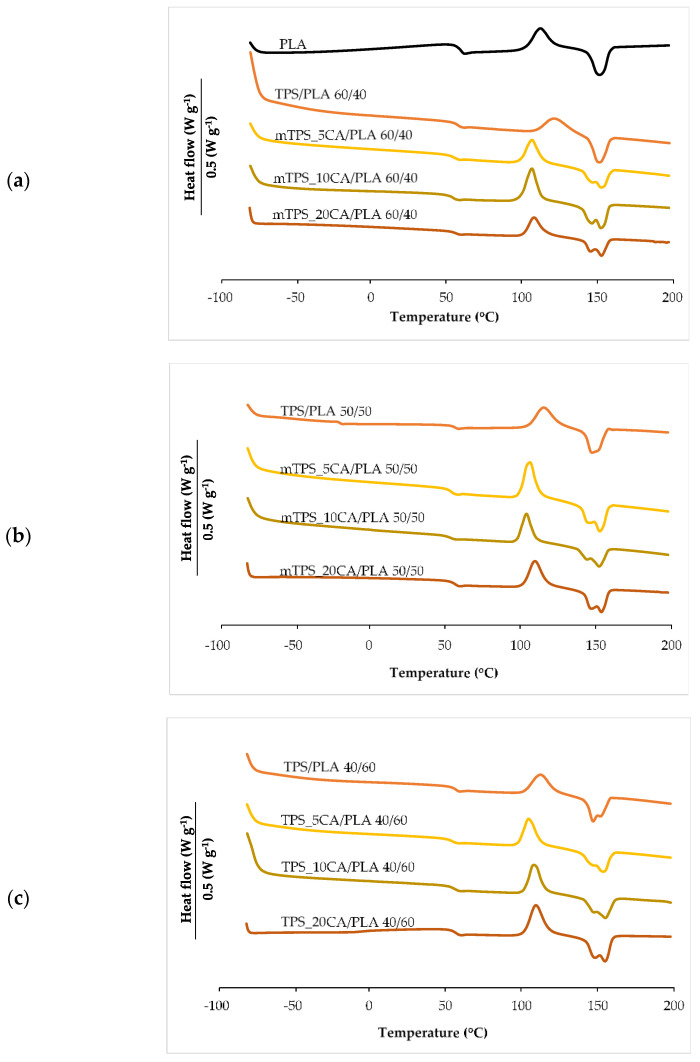
DSC thermograms of (**a**) PLA, TPS/PLA, and mTPS/PLA 60/40; (**b**) TPS/PLA and mTPS/PLA 50/50; and (**c**) TPS/PLA and mTPS/PLA 40/60 biopolymeric blends.

Differential scanning calorimetry (DSC) was used to investigate the influence of TPS with and without CA as a compatibilizer on the thermal transitions of PLA (Figure 4, Table 4). The second heating cycle was analyzed to determine the glass transition temperature (*T_g_*), the cold crystallization temperature (*T_cc_*), the melting temperature (*T_m_*), and the corresponding enthalpies (Δ*H_cc_* and Δ*H_m_*). The degree of crystallinity (*χ_c_*) was calculated according to the Equation (1). The DSC thermogram of pure PLA showed characteristic transitions: *T_g_* at 58.5 °C, *T_cc_* at 112.4 °C, and *T_m_* at 152.1 °C, in agreement with literature data (Figure 4a) [34,47]. After incorporation of TPS, a decrease in *T_g_* of about 2–3 °C was observed, indicating increased chain mobility and improved miscibility between PLA and TPS phases [10]. Increasing the TPS content did not lead to a further significant decrease in *T_g_*. However, the addition of CA led to a more significant decrease in *T_g_*, indicating an improved interfacial interaction due to potential esterification reactions between PLA carboxylic acid and starch hydroxyl groups. CA also strongly influenced the cold crystallization behavior of PLA. Blends containing CA showed lower *T_cc_* values compared to those without CA, especially in the mTPS_10CA/PLA 50/50 blend (Table 4), where the *T_cc_* decreased to 104.4 °C. This indicates that CA accelerates the crystallization of PLA during heating and likely acts as a compatibilizer that promotes nucleation. TPS, which is completely amorphous, serves as a physical nucleating agent that facilitates the reorganization of the macromolecular PLA chains into crystalline domains. The thermal curves of TPS/PLA polymer blends showed a remarkable change in melting behavior. While pure PLA showed a single melting peak, several TPS/PLA blends, especially those with CA, showed double melting peaks. This behavior is typical for PLA and is associated with the presence of imperfect crystals or dual crystalline phases. One explanation attributes the double melting peaks to the coexistence of α- and β-crystal forms, with the α-form melting at a higher temperature and being thermodynamically more stable, while the β-form, which is less ordered, melts at a lower temperature [48,49]. Alternatively, the double peak may result from the process of melting, recrystallization, and remelting during heating, in which less ordered crystals melt, recrystallize into more stable structures, and then melt again at a higher temperature. Interestingly, in TPS/PLA blends with 60/40 (Figure 4a) and 50/50 compositions, the double melting peak was not pronounced, suggesting that the presence of TPS alters the crystal formation kinetics of PLA. A general decrease in Tm was observed in the blends compared to pure PLA, reflecting the dilution and morphological effects of amorphous TPS. Thermodynamically, this decrease can be attributed to the reduction of the chemical potential of the crystalline phase of PLA due to the incorporation of the amorphous component. However, the presence of citric acid counteracted this trend. The blend mTPS_20CA/PLA 60/40 had the highest *T_m_* (155.0 °C), while the blend TPS/PLA 40/60 (Table 4) without CA had the lowest (147.0 °C). This indicates that CA promotes higher crystallinity and thermal ordering within the PLA matrix. An analysis of the degree of crystallinity further supported these observations. TPS significantly improved PLA crystallization and acted as a nucleating agent. The addition of 5 wt.% CA increased the *χ_c_* value in all blend ratios, while higher CA contents led to a decrease in crystallinity, except for the mTPS_10CA/PLA blend, which reached the highest *χ_c_* value of 58.0%. At higher CA concentrations, the PLA matrix becomes softer due to increased chain mobility; however, this also hinders the regular alignment and packing required for efficient crystallization. Excessive CA concentration can also disrupt phase compatibility, leading to phase separation and lower crystallization efficiency, thus promoting an amorphous structure. In summary, the thermoplastic starch of the Scala variety significantly changes the thermal properties of PLA, especially in combination with citric acid. The optimal compatibilization effect was achieved with 10 wt.% CA, which increased crystallinity and promoted more efficient cold crystallization. These findings are relevant for the development of biodegradable PLA-based materials with tailored thermal properties for specific applications, such as sustainable packaging.

### 3.6. Thermogravimetric Analysis of Biopolymeric Blends

**Figure 5:**
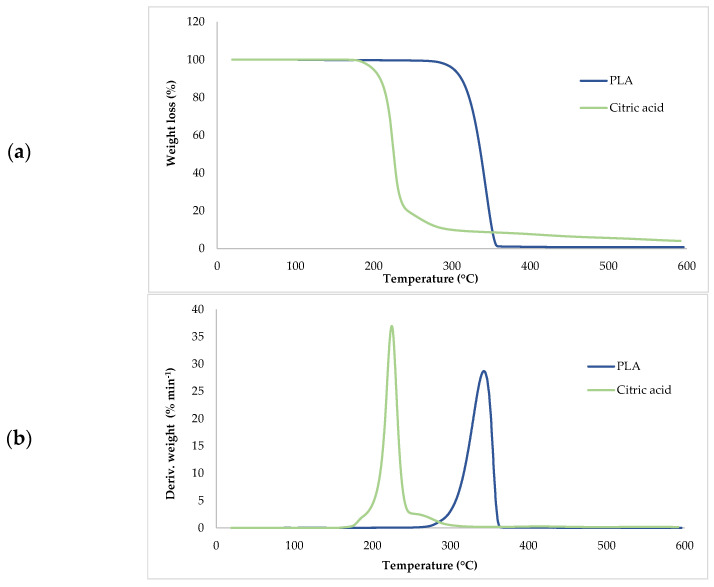
(**a**) TG; (**b**) DTG curves of PLA and citric acid.

**Figure 6:**
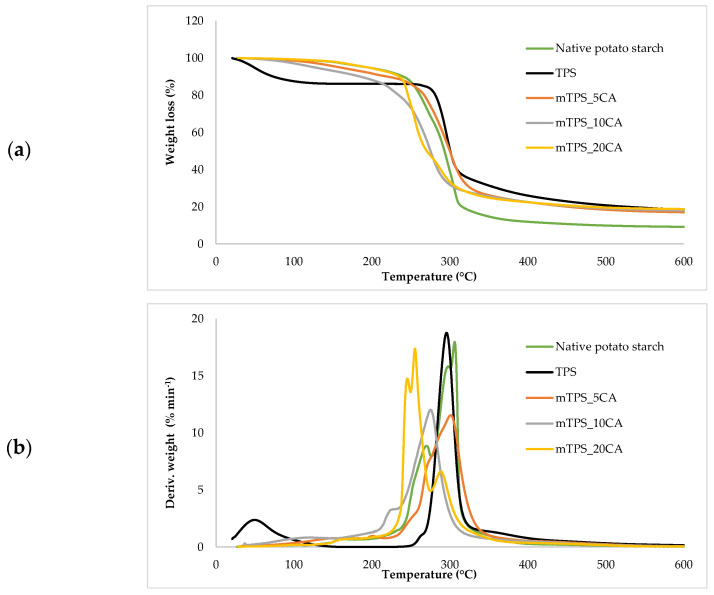
(**a**) TG; (**b**) DTG curves of native potato starch, TPS, and mTPS.

**Figure 7:**
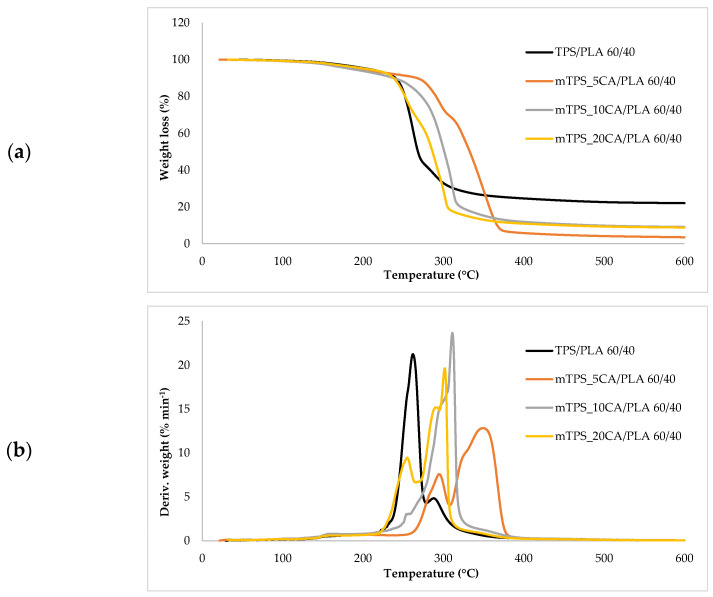
(**a**) TG; (**b**) DTG curves TPS/PLA and mTPS/PLA 60/40 biopolymeric blends.

**Figure 8:**
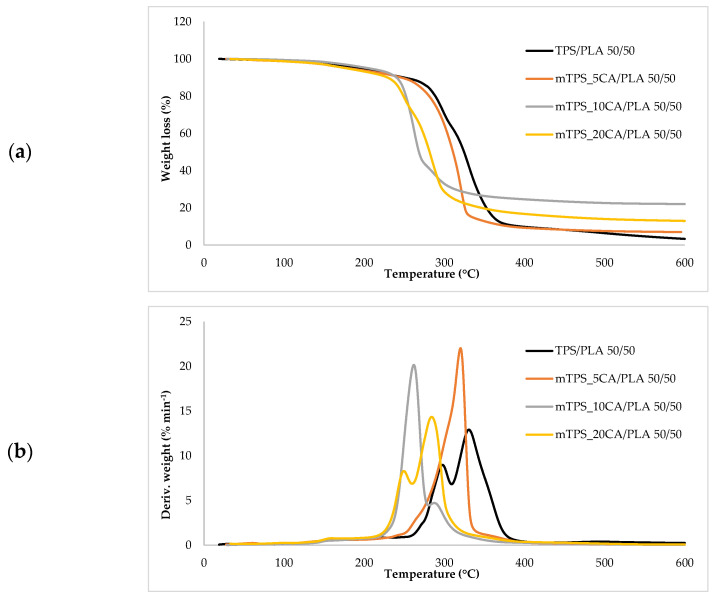
(**a**) TG; (**b**) DTG curves TPS/PLA and mTPS/PLA 50/50 biopolymeric blends.

**Figure 9:**
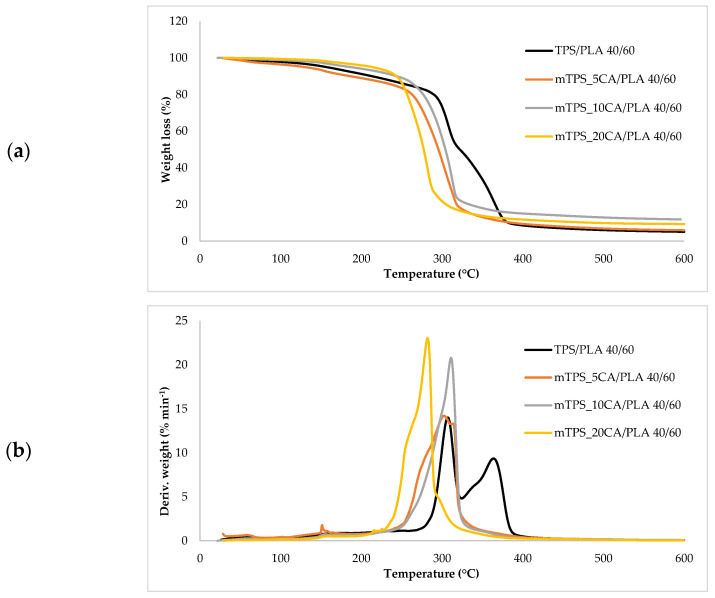
(**a**) TG; (**b**) DTG curves TPS/PLA and mTPS/PLA 40/60 biopolymeric blends.

Thermogravimetric analysis (TGA) was used to investigate the thermal stability of native potato starch, pure TPS of the Scala variety and TPS/PLA biopolymeric blends with and without CA as a compatibilizer. Since biodegradable materials usually degrade at lower temperatures compared to synthetic polymers, the determination of their thermal behavior is crucial for the evaluation of processing conditions and potential applications in thermally demanding environments [50]. The thermal stability of the samples was characterized based on the initial degradation temperature (*T_onset_*), the maximum degradation temperatures (*T_max1_* and *T_max2_*) corresponding to the peak degradation rates (from DTG curves), the final degradation temperature (*T_end_*) and the residual mass at 600 °C (*R_600_*_°C_). The relevant thermal parameters are listed in Table 5., and representative TG and DTG curves are shown in Figure 5, Figure 6, Figure 7, Figure 8 and Figure 9. The initial weight loss at ≤=100 °C is associated with the evaporation of free, physically adsorbed water for all samples, including native starch and TPS. The lower mass loss observed for mTPS_XCA samples indicates reduced moisture uptake, likely due to improved structural organization and CA-induced hydrophobicity (Figure 6). When the temperature rises above 100 °C, further weight loss is attributed to the release of more strongly bound water molecules, which are connected to the starch matrix via hydrogen bonds. This phase is more pronounced in samples containing glycerol, as glycerol interrupts the starch–starch interactions and binds water more efficiently. TPS shows a remarkable weight loss around 50.6 °C (Figure 5a), reflecting the simultaneous release of free and bound water as well as an early softening of the matrix due to glycerol plasticization. The main degradation phase takes place between 270 and 330 °C and is characterized by the cleavage of the glycosidic bonds and the depolymerization of the polysaccharide chains. This process leads to the formation of low-molecular-weight products such as acetic acid and furfural, especially in native starch, as indicated by several peaks in the DTG curves. These volatile degradation products can impair the stability of the material in the subsequent processing stages. TPS exhibited a higher onset temperature for degradation compared to native starch, indicating improved thermal stability due to the plasticization effect and more ordered molecular packing. The use of CA as a compatibilizer further alters this behavior. The addition of CA leads to a reduction in intermolecular hydrogen bonding and promotes the formation of more amorphous domains. At lower concentrations, CA improves thermal stability, while higher concentrations can increase the acidity of the medium, favoring degradation processes such as hydrolysis and esterification [19,31,36]. These processes manifest themselves in the form of several degradation peaks, as can be seen in Figure 5a,b PLA and TPS/PLA blends. Pure PLA underwent a one-step degradation process in the narrow temperature range of 287–363 °C, with *T_max2_* at 345 °C, and exhibited a mass loss of 98.5%, leaving about 1% residue. This behavior is consistent with literature data and is attributed to the cleavage of end chains within the PLA macromolecules [47]. In contrast, TPS/PLA blends exhibited lower *T_onset_* values than pure PLA, confirming lower thermal stability due to TPS incorporation. The degradation profile of these blends showed two different stages: the first corresponds to the degradation of the TPS, the second to the degradation of the PLA. This becomes particularly clear in Figure 7a,b, where TPS/PLA 60/40 blends with and without CA are compared. The shoulder observed at approximately 294 °C in the mTPS_10CA/PLA 60/40 sample indicates improved compatibility and stronger interfacial interactions between the two polymer phases, possibly due to esterification reactions promoted by CA. Interestingly, the addition of 5 wt.% CA resulted in the most significant changes in the degradation parameters. Compared to the unmodified blend, mTPS_5CA/PLA 60/40 showed increased *T_max_* values and a delayed onset of degradation, indicating improved thermal stability. On the other hand, the blend with 20 wt.% CA exhibited the least shift, possibly due to excessive acidity and catalyzed hydrolytic degradation. However, the blend with 20% CA points to biphasic behavior, i.e., phase separation. These observations are consistent with the dual role of CA as a plasticizer and as a degradation promoter at higher concentrations [51]. The residual mass at 600 °C increased with increasing TPS and CA content, probably due to the presence of non-volatile inorganic components such as ash, salts, or CA-derived residual compounds [52]. This trend is evident in Figure 5a,b, where blends with 10 and 20 wt.% CA have a higher residue content than the pure polymers. In addition, the longer degradation interval observed in TPS/PLA blends compared to the pure components indicates a more gradual thermal decomposition mechanism, especially for systems with a higher TPS content. In summary, the thermal decomposition of TPS/PLA blends proceeds in two distinct phases, reflecting the sequential degradation of TPS and PLA phases. The thermal stability of these systems is influenced by both the composition and the compatibilizer content: The incorporation of TPS reduces the thermal stability of PLA but promotes a broader, more controlled degradation behavior. The addition of CA at 5 wt.% improves the initial and maximum degradation temperatures, indicating optimal compatibilization and increased resistance to thermal degradation. Higher CA concentrations (10–20 wt.%) contribute to increased residue formation and possibly catalyze decomposition by acid-induced hydrolysis. These results underline the importance of optimizing the compatibilizer content to achieve a balance between thermal processability and material stability in biodegradable starch-based polymer blends.

### 3.7. Water Vapor Permeability (WVP) of the Biopolymeric Blends

**Figure 10:**
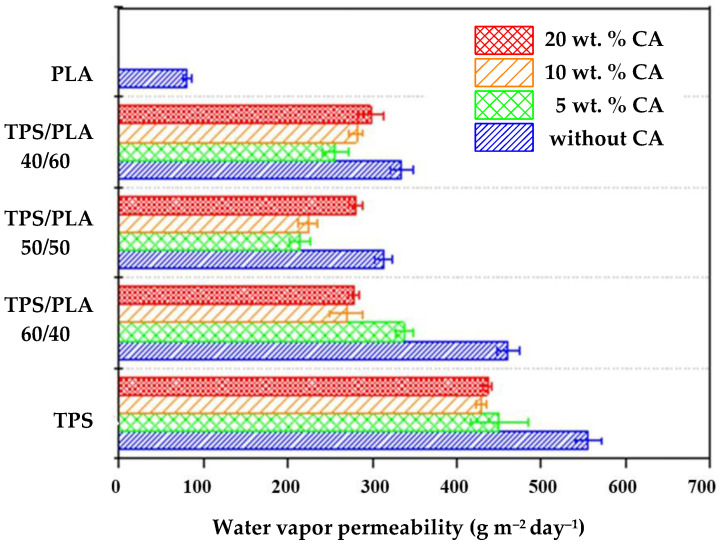
Water vapor permeability of biopolymeric blends.

Figure 10 shows the WVP results for biodegradable polymer blends based on TPS and PLA, with and without citric acid modification. Due to its hydrophobic nature, pure PLA has a low WVP of 80.435 g m^−2^day^−1^. In contrast, unmodified TPS has a significantly higher permeability of 554.545 g m^−2^day^−1^, which is consistent with the hydrophilic nature of the starch. The WVP values of all TPS/PLA blends lie between the two extreme values of the pure components. The lowest permeability is observed for the mTPS_5CA/PLA 50/50 blend, which is probably due to the optimal balance between the hydrophobic PLA and modified TPS phases. On the other hand, the highest permeability is observed with mTPS_5CA/PLA 60/40, which is due to a higher proportion of hydrophilic TPS. Interestingly, in contrast to the results for other starch sources, Scala-based blends do not show a consistent downward trend in WVP with increasing CA content. The addition of 5 wt.% CA decreases permeability, which is due to partial esterification between the hydroxyl groups of the starch and the carboxyl groups of the citric acid. This crosslinking reduces the chain mobility and the free volume, which improves the barrier properties [9,10,36,42]. However, further increasing the citric acid content (10 and 20 wt.%) leads to higher WVP values. This is probably due to the following: excess unreacted citric acid acts as a plasticizer and increases the flexibility and permeability of the matrix and possibly acid-catalyzed hydrolysis of glycosidic bonds, resulting in reduced molecular weight and increased porosity [19,31,32,42]. These results suggest an optimal CA content of ~5 wt.% to improve WVP performance in TPS/PLA blends.

### 3.8. Water Absorption of the Biopolymeric Blends

**Figure 11:**
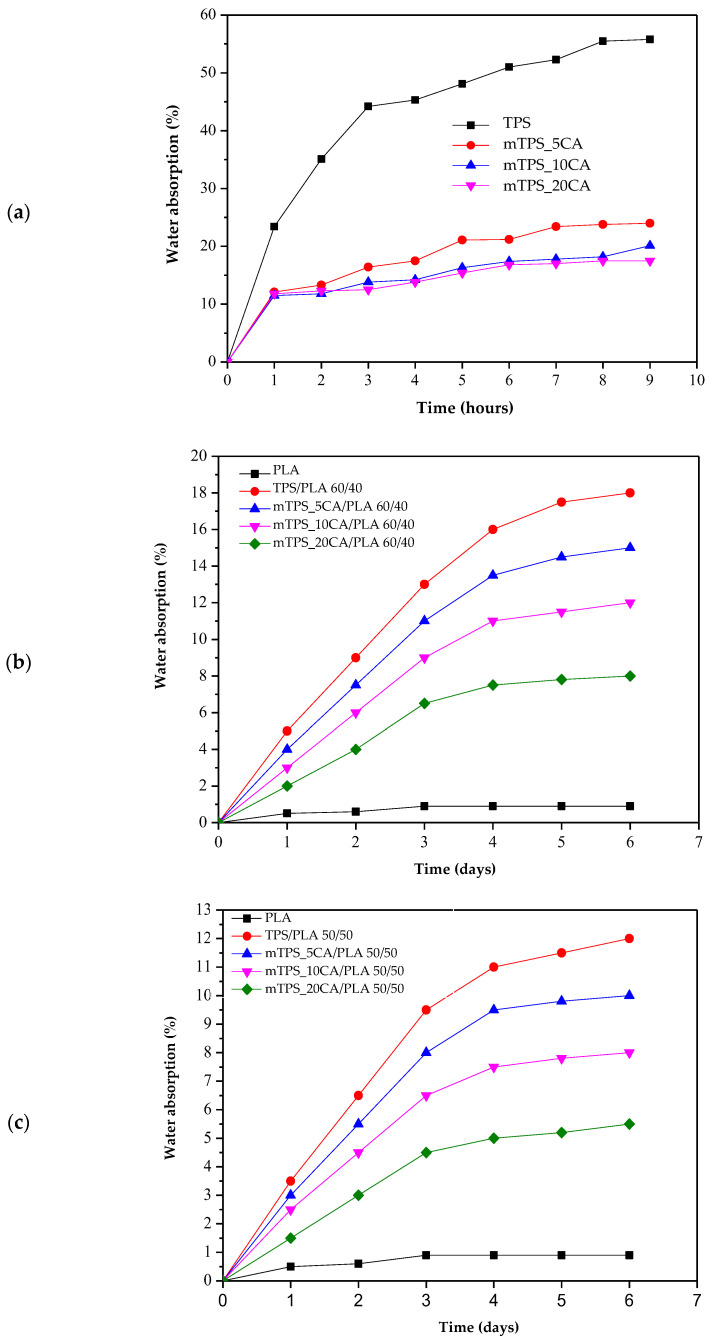

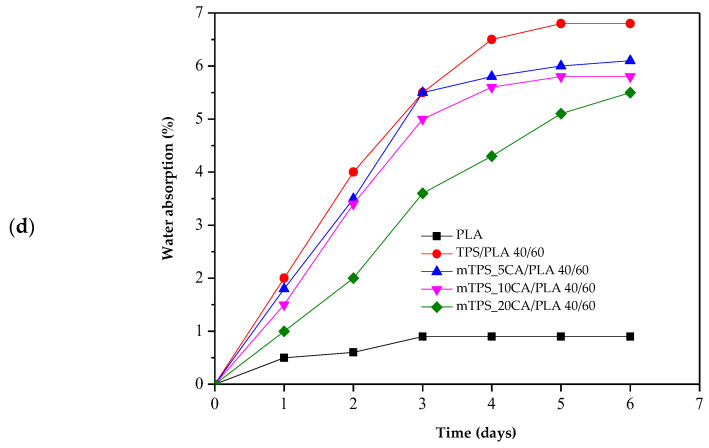
Water absorption of (**a**) TPS and mTPS; (**b**) PLA, TPS/PLA, and mTPS/PLA 60/40; (**c**) PLA, TPS/PLA, and mTPS/PLA 50/50; and (**d**) PLA, TPS/PLA, and mTPS/PLA 40/60 biopolymeric blends.

Due to its high hydrophilicity, TPS absorbs water rapidly and to a significant extent, resulting in swelling and structural degradation. All pure and modified mTPS samples (5, 10, and 20 wt.% CA) disintegrated within 24 h, making long-term measurements impractical. Therefore, water absorption was monitored over 10 h, and the results are shown in Figure 11a. The results confirm that the chemical modification of mTPS with citric acid effectively reduces water absorption. The formation of ester bonds between the citric acid and the hydroxyl groups of the starch leads to a crosslinked structure with reduced water permeability. However, no significant difference is observed between the samples modified with 5, 10, and 20 wt.% CA, indicating that the improvement in water resistance reaches a plateau beyond a certain crosslinking threshold. The water absorption of TPS/PLA blends over 6 days is shown in Figure 11b–d. As expected, pure PLA shows the lowest water absorption, which is consistent with its hydrophobic property. A gradual increase in water absorption can be observed for all blends with increasing TPS content, particularly for the TPS/PLA composition 60/40, which has the highest TPS content. The citric acid modification of TPS in these blends results in lower water absorption, which is due to improved hydrophobicity and the formation of a more compact, crosslinked matrix. Additionally, higher PLA content leads to lower water absorption in all compositions, highlighting the role of PLA in forming a protective continuous phase that limits the exposure of TPS to moisture. Conversely, a higher TPS content leads to more voids and hydrophilic domains, which increases water absorption. These observations are consistent with previous studies [10,42,53] and confirm that citric acid effectively improves the performance of TPS-based materials under humid conditions up to a saturation point, beyond which further addition may not provide significant benefits.

### 3.9. Mechanical Properties of the Biopolymeric Blends

**Figure 12:**
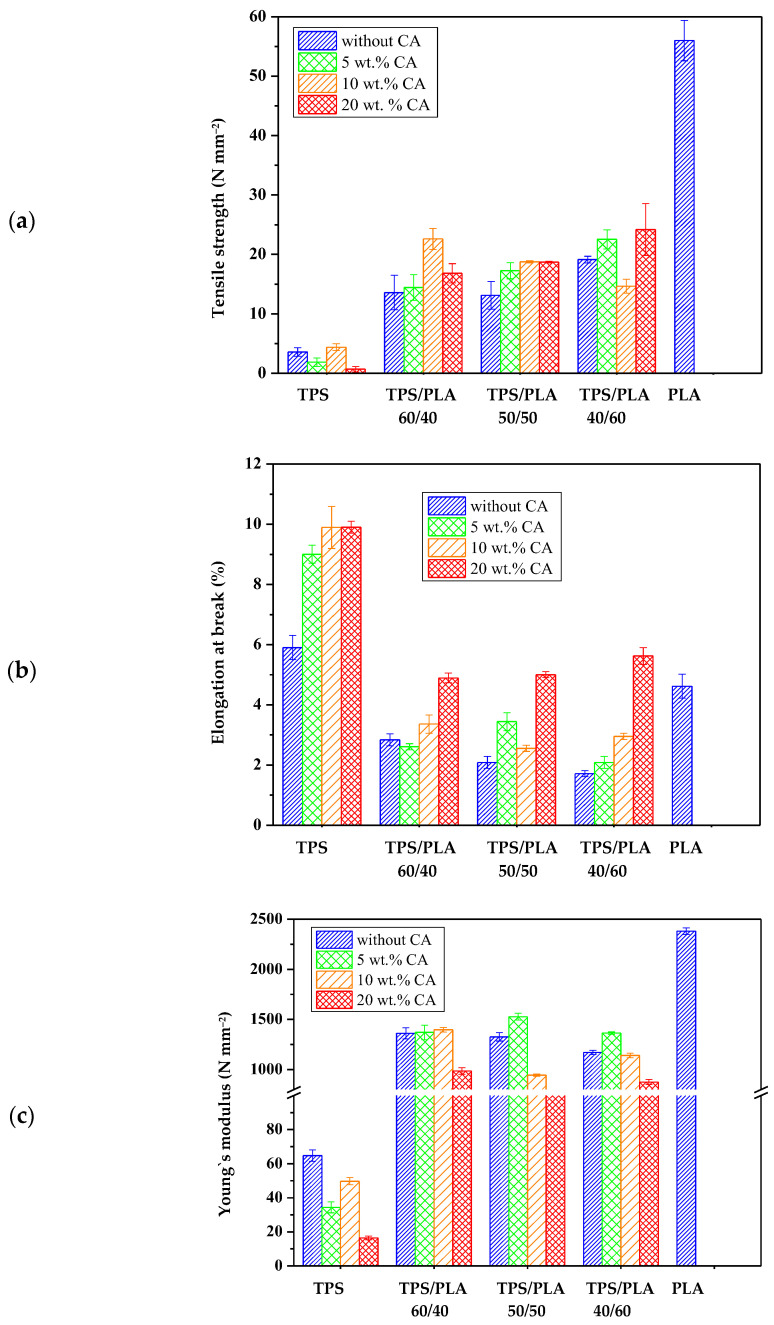
Mechanical properties of biopolymeric blends: (**a**) tensile strength; (**b**) elongation at break; (**c**) Young’s modulus.

The mechanical properties of polymer blends are crucial for their potential use in packaging, especially for biodegradable alternatives [54]. Figure 12 show the characteristic values of tensile strength (N mm^−2^), elongation at break (%), and modulus of elasticity (N mm^−2^) for samples of pure PLA, TPS, and TPS/PLA blends in the ratios 60/40, 50/50, and 40/60, each with and without citric acid modification. Pure PLA has the highest tensile strength and modulus of elasticity, reflecting its rigid and brittle nature. Conversely, pure TPS has the lowest tensile strength and modulus but has a slightly higher elongation at break, which is due to its flexible but mechanically weak character. The mechanical properties of TPS/PLA blends lie between the values of the pure components, confirming that the blend increases the strength and stiffness of TPS while slightly reducing the inherent brittleness of PLA. However, the extent of the mechanical improvement depends on the blend ratio and the presence of citric acid as a modifier. The addition of CA has a limited but recognizable effect on the mechanical properties. For most compositions, no consistent trend towards increased flexibility or strength was observed with increasing citric acid content, except for the TPS/PLA 40/60 blend, where a significant increase in elongation at break was observed. This suggests that the lower PLA content allows for greater expression of the plasticizer and crosslinking effects induced by citric acid. These mechanical changes are probably associated with the dual role of CA: as a crosslinking agent, citric acid can form ester bonds with starch, which slightly improves mechanical integrity and interfacial compatibility with PLA, and as a plasticizer, it can reduce intermolecular forces and increase chain mobility, particularly in starch-rich blends. While the overall effects were moderate, the polymer blends based on the TPS Scala variety exhibited noticeable improvements, particularly in formulations with higher starch content, where crosslinking induced by citric acid is likely to play a dominant role. The morphological properties of these blends, such as phase adhesion and dispersion, probably contribute to the observed mechanical behavior. Improved compatibility at the interface between modified TPS and PLA may enable more efficient stress transfer and thus improve mechanical performance. While this effect is not as pronounced as in other systems, it is present to a modest extent, particularly in blends where the TPS phase is sufficiently dispersed and partially crosslinked. In summary, TPS/PLA blends have mechanical properties suitable for flexible packaging and can be optimized by modification with citric acid, particularly in starch-rich formulations. However, the effect of modification seems to be more composition dependent for the Scala grade compared to other starch types.

### 3.10. Biodegradability of the Biopolymeric Blends

**Figure 13:**
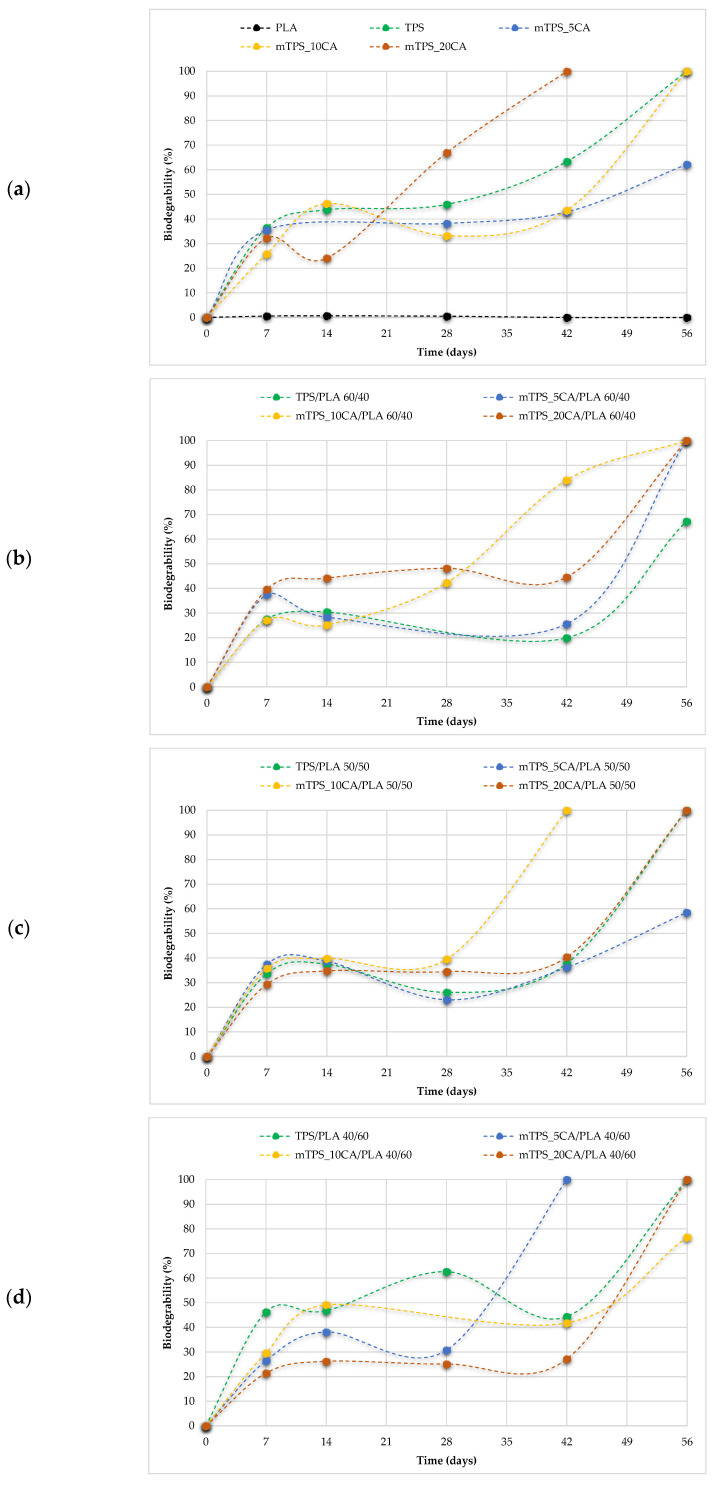
Biodegradability of (**a**) PLA, TPS, and mTPS; (**b**) TPS/PLA and mTPS/PLA 60/40; (**c**) TPS/PLA and mTPS/PLA 50/50; and (**d**) TPS/PLA and mTPS/PLA 40/60 biopolymeric blends.

**Figure 14:**
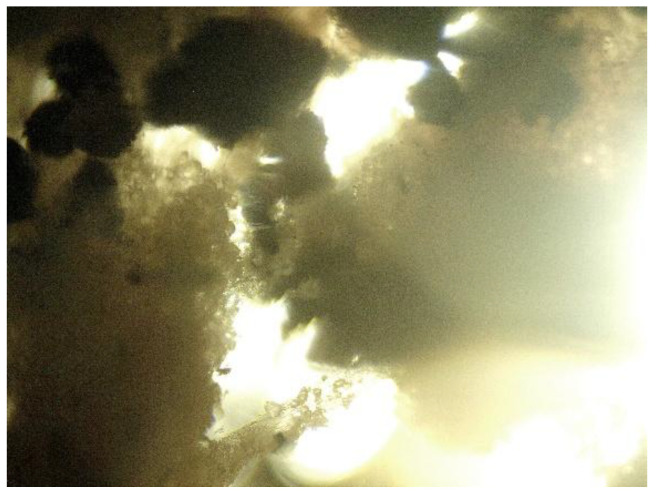
Porous areas of thermoplastic starch, photographed on the 14th day of biodegradation with a light microscope (400×).

**Figure 15:**
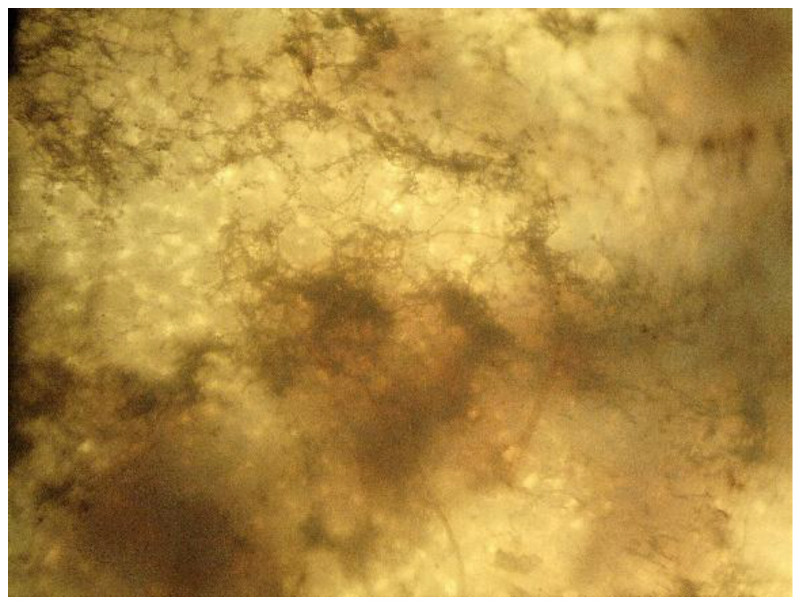
Microphotograph of mold on the sample mTPS_20CA/PLA 50/50, taken on the 28th day of biodegradation with a light microscope (400×).

Biodegradability is a key parameter in assessing the environmental compatibility of biodegradable polymer materials. During the 56-day study period, all polymer TPS/PLA blends as well as the pure components were biodegraded to varying degrees depending on their composition and CA content. As shown in Figure 13a, pure TPS and mTPS_10CA showed complete degradation within the 56-day period. mTPS_20CA was completely degraded after 42 days, while the sample with 5 wt.% CA was already 61% degraded after 56 days. These results indicate that the CA content influences the biodegradability, with an addition of 10–20 wt.% accelerating degradation and lower concentrations (5 wt.%) having a retarding effect. Figure 14 provides visual evidence of the biodegradation process in TPS. The observed heterogeneous porosity distribution across the sample surface suggests non-uniform degradation, which is likely due to variations in water absorption and enzymatic activity [55,56]. These differences may be due to the local ratio of amylose to amylopectin as well as the degree of gelatinization achieved during processing. Amylose-rich regions, with their linear structure, tend to form stronger gels and degrade more slowly, while amylopectin, which is more branched and hydrophilic, allows for faster degradation. The importance of processing parameters, particularly extrusion conditions and starch modification, is thus emphasized, as they directly affect the microstructure and biodegradation kinetics of TPS-based materials. The biodegradability of TPS/PLA blends also varied with the blend ratio and CA content. As shown in Figure 13, all biopolymeric blends with a TPS/PLA 60/40 were completely degraded within 56 days, regardless of CA content. Interestingly, the blend without CA was only 68% degraded, indicating that citric acid promotes biodegradation in this formulation. For TPS/PLA 50/50 blends, complete degradation was observed by day 42 for the sample mTPS_10CA/PLA 50/50, while blends without CA and those with 20 wt.% CA were completely degraded by day 52. The blend with 5 wt.% CA by weight was incompletely degraded at the end of the test period (after 56 days). A similar trend was observed for TPS/PLA 40/60 blends, where samples modified with citric acid generally showed faster or more complete degradation, although the effect was less uniform. For example, the 10 wt.% CA blend was completely degraded by day 42, while the 5 wt.% sample was only 58% degraded by day 56. The positive effect of citric acid on biodegradation can be explained by several mechanisms: With plasticization and crosslinking, citric acid forms ester bonds with the hydroxyl groups of starch, which improves interfacial compatibility but also alters hydrophilicity. With increased microstructural degradation, CA may lead to more accessible amorphous areas, which promote microbial attack, particularly in starch-rich mixtures. With local acidification, the carboxyl groups of citric acid may lower the pH, selectively promoting or inhibiting certain microbial communities (Figure 15).

**Figure 16:**
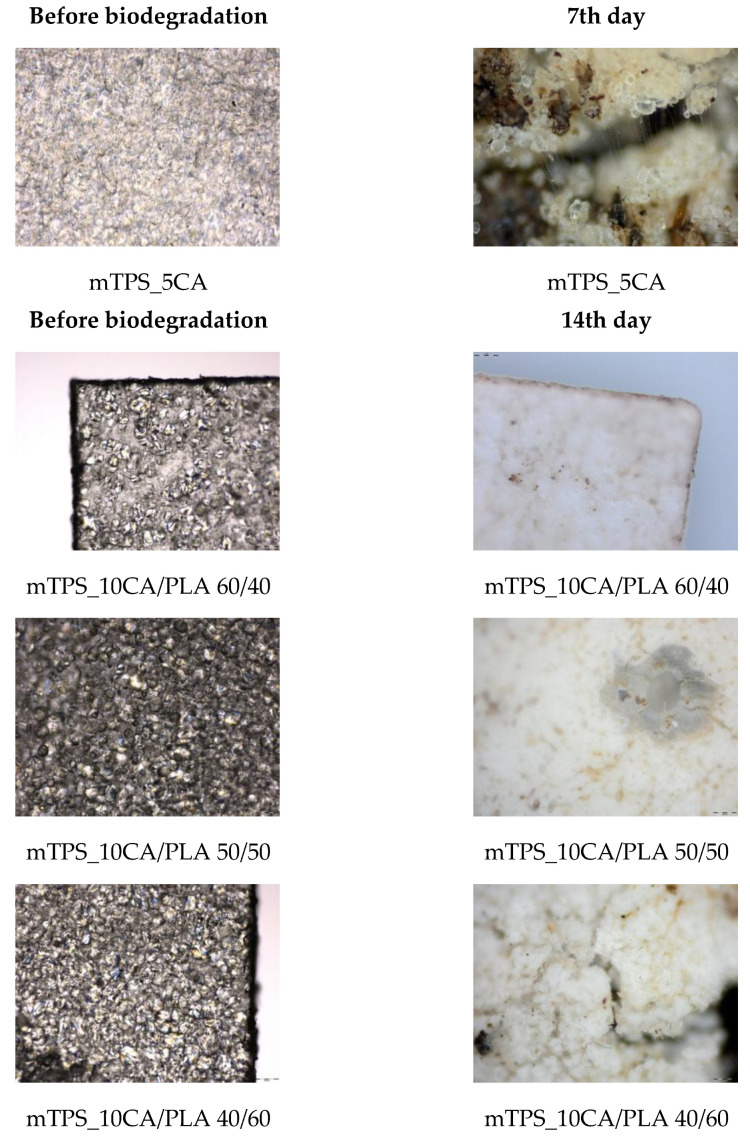
Surface microstructure of mTPS/PLA biopolymeric blends before and during biodegradation obtained with polarizing optical microscope (100×).

**Figure 17:**
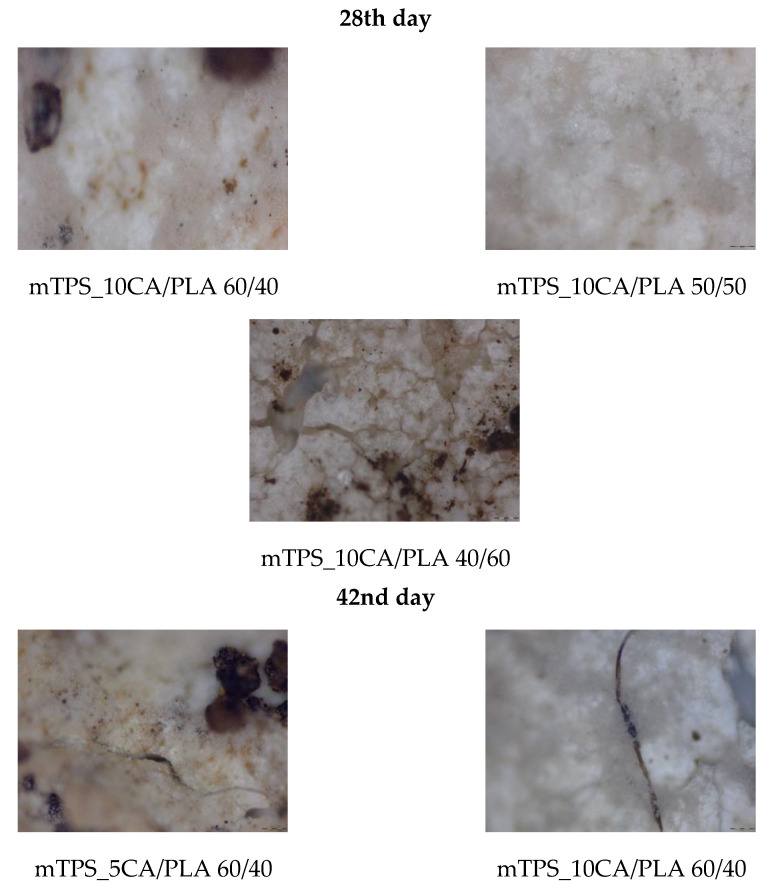
Surface microstructure of mTPS/PLA biopolymeric blends after 28th and 42nd day of biodegradation obtained with polarizing optical microscope (100×).

**Figure 18:**
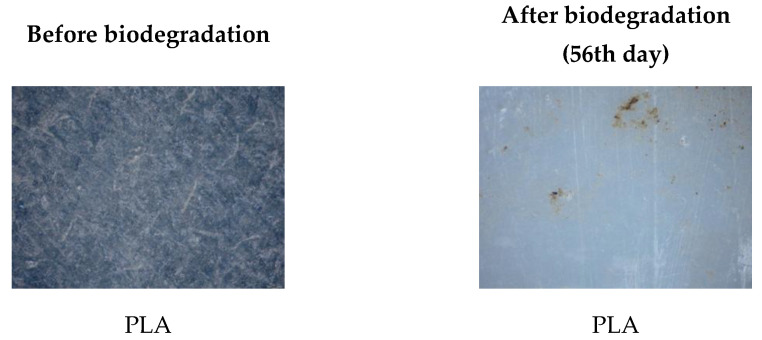
Surface microstructure of pure PLA before and after 56th day of biodegradation obtained with polarizing optical microscope (100×).

**Figure 19:**
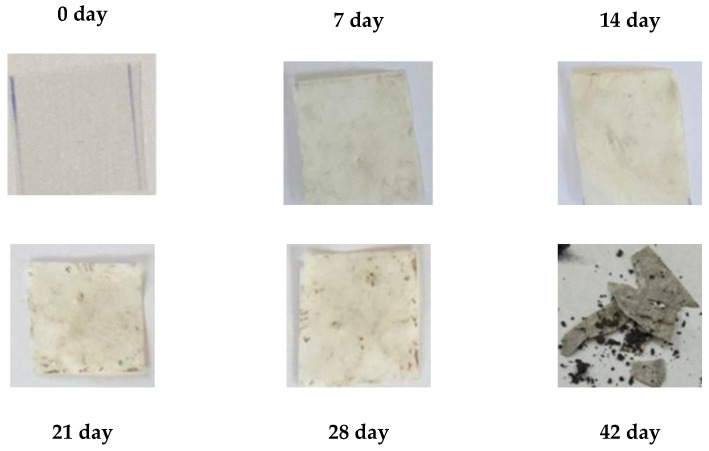
Biodegradation of pure PLA.

However, excessive crosslinking or reduced swelling capacity, especially at lower citric acid concentrations, may limit microbial access, which explains the slower degradation in the 5 wt.% samples. The microscopic analysis (Figure 16 and Figure 17) confirms the progressive structural degradation of the TPS/PLA blends. In the early stages (day 7), signs of surface erosion and microbial colonization can be seen. On day 14, the blends, especially those with 10 wt.% CA, show increasing fragmentation and roughened surfaces, indicating advanced biodegradation. By day 28, the samples show extensive degradation, and by day 42, the surfaces are highly porous and fractured, indicating a breakdown in the integrity of the material. These microscopic images confirm the hypothesis that citric acid modification accelerates degradation, especially in well-dispersed, amorphous TPS domains. In contrast, pure PLA remained structurally intact throughout the 56-day period (Figure 18 and Figure 19) and showed no signs of degradation or microbial colonization, which is consistent with previous studies [57]. SEM micrographs (Figure 2) confirm the smooth, unaltered surface of PLA and emphasize the need for blending with biodegradable fillers such as TPS to improve environmental compatibility. The biodegradability of TPS and TPS/PLA blends is strongly influenced by the composition and the citric acid content. While pure TPS degrades quickly, the degradation rate is reduced by the blend with PLA, depending on the blend ratio and degree of modification. CA generally promotes biodegradation in mTPS_XCA-based blends, especially at 10 and 20 wt.%, although the effect may vary due to complex interactions between material structure, hydrophilicity, and microbial accessibility. These results highlight the potential of TPS/PLA blends as biodegradable alternatives in packaging, where the rate of degradation is adjustable depending on the formulation and modification strategy.

## 4. Conclusions

This study offers a comprehensive evaluation of biodegradable polymer blends based on Scala potato thermoplastic starch and its combinations with polylactic acid, with and without citric acid as a reactive compatibilizer. The findings confirm that Scala starch, derived from a locally cultivated potato variety, is a valuable raw material for sustainable polymer development, meeting the requirements of purity, processability, and functionality necessary for packaging applications. The results demonstrate that the addition of citric acid to Scala-based starch significantly enhances material performance by improving mechanical strength, thermal stability, and moisture resistance while also promoting better compatibility with PLA. FTIR analysis confirmed the formation of ester bonds, indicating partial crosslinking and structural modification, which in turn resulted in improved interfacial adhesion and more homogeneous blends. Thermal and morphological analyses further revealed that citric acid acts not only as a natural compatibilizer but also as a plasticizing and crosslinking agent, enabling the fine-tuning of blend properties. However, its effect on the crystalline organization of PLA remains limited, and formulations with starch contents above 60% still exhibited phase separation, highlighting the importance of carefully balancing blend ratios. From an application perspective, these findings are highly relevant for the development of next-generation biodegradable packaging materials. The studied blends exhibit faster biodegradation than pure PLA, reduced water sensitivity compared to unmodified starch systems, and improved barrier and mechanical performance, making them particularly attractive as eco-friendly alternatives to petroleum-based plastics. Importantly, this work extends beyond many existing studies by integrating the entire chain of material development, from starch isolation and chemical modification to blend design, performance evaluation, and biodegradation assessment, thereby providing a holistic understanding of how raw material origin, chemical modification, and formulation strategy collectively determine end-use properties. Overall, this study underscores the potential of citric acid-modified Scala starch/PLA blends as innovative, renewable, and sustainable materials for food packaging applications. At the same time, it highlights the need for ongoing interdisciplinary efforts, particularly in the areas of life cycle assessment (LCA), industrial processing optimization, and scaling strategies, to ensure that such biodegradable solutions are not only scientifically sound but also economically and environmentally viable within a circular economy framework.

## Figures and Tables

**Table 1:** Properties of the starch obtained from the Scala potato variety [24].

Starch from Scala Variety	
**Chemical composition % d.m.**	
Dry Matter	84.95 ± 0.01
Protein	0.09 ± 0.00
Fat	0.01 ± 0.00
Ash	0.25 ± 0.00
Starch	82.95 ± 0.42
Crude Fiber	0.90 ± 0.01
Amylose	16.97 ± 0.37
**Color properties**	
L*	94.18 ± 0.25
a*	−1.32 ± 0.02
b*	2.31 ± 0.07
**Gelatinization parameters**	
*T_o_* (°C)	67.52 ± 0.59
*T_p_* (°C)	71.41 ± 0.06
*T_e_* (°C)	77.05 ± 0.17
∆*H* (J g^−1^)	2.30 ± 0.04
**Pasting properties**	
Peak Viscosity [BU]	1673.5 ± 20.5
Viscosity at 92 °C [BU]	974.0 ± 9.9
Viscosity at 50 °C [BU]	915.0 ± 4.2
**Texture properties of starch gels**	
Gel Strength (g)	3.03 ± 0.08
Rupture Strength (g)	912.11 ± 17.51
Adhesiveness (g sec)	−192.56 ± 5.68

Color coordinates: L* represents lightness from black (0) to white (100), a* indicates the red (positive)-green (negative) axis, b* indicates the yellow (positive)-blue (negative) axis.

**Table 2:** Composition and labeling of biopolymeric blends.

Samples	TPS (wt.%)	PLA (wt.%)	mTPS_5CA (wt.%)	mTPS_10CA (wt.%)	mTPS_20CA (wt.%)
PLA	-	100	-	-	-
TPS	100	-	-	-	-
mTPS_ 5CA	-	-	100	-	-
mTPS_ 10CA	-	-	-	100	-
mTPS_ 20CA	-	-	-	-	100
TPS/PLA 60/40	60	40	-	-	-
TPS/PLA 50/50	50	50	-	-	-
TPS/PLA 40/60	40	60	-	-	-
mTPS_5CA/PLA 60/40	-	40	60	-	-
mTPS_5CA/PLA 50/50	-	50	50	-	-
mTPS_5CA/PLA 40/60	-	60	40	-	-
mTPS_10CA/PLA 60/40	-	40	-	60	-
mTPS_10CA/PLA 50/50	-	50	-	50	-
mTPS_10CA/PLA 40/60	-	60	-	40	-
mTPS_20CA/PLA 60/40	-	40	-	-	60
mTPS_20CA/PLA 50/50	-	50	-	-	50
mTPS_20CA/PLA 40/60	-	60	-	-	40

**Table 3:** Melt flow index (MFI) of biopolymeric blends.

Sample	MFI (g(10 min)^−1^)
PLA	6.3 ± 0.7
TPS/PLA 60/40	2.2 ± 0.1
TPS/PLA 50/50	4.5 ± 0.9
TPS/PLA 40/60	5.1 ± 1.1
mTPS_5CA/PLA 60/40	2.2 ± 0.3
mTPS_5CA/PLA 50/50	5.0 ± 0.8
mTPS_5CA/PLA 40/60	4.9 ± 0.4
mTPS_10CA/PLA 60/40	1.7 ± 0.2
mTPS_10CA/PLA 50/50	3.7 ± 0.3
mTPS_10CA/PLA 40/60	5.2 ± 0.8
mTPS_20CA/PLA 60/40	2.1 ± 0.1
mTPS_20CA/PLA 50/50	4.9 ± 0.3
mTPS_20CA/PLA 40/60	5.3 ± 0.1

**Table 4:** Thermal properties of biopolymeric blends.

Sample	*T_g_* (°C)	*T_c_* (°C)	Δ*H_c_* (Jg^−1^)	*T_m_* (°C)	Δ*H_m_* (Jg^−1^)	*χ_c_* (%)
PLA	58.5	112.4	20.9	152.1	20.2	21.7
TPS/PLA 60/40	56.8	122.0	16.6	151.4	17.6	47.3
TPS/PLA 50/50	56.3	115.6	18.7	148.0	15.4	33.1
TPS/PLA 40/60	56.0	112.7	16.2	147.0	15.0	26.9
mTPS_5CA/PLA 60/40	54.7	106.9	17.1	153.6	17.9	48.1
mTPS_5CA/PLA 50/50	53.8	106.2	21.7	152.9	21.2	45.5
mTPS_5CA/PLA 40/60	53.6	105.0	15.9	153.7	16.8	30.1
mTPS_10CA/PLA 60/40	53.3	106.9	22.7	153.4	21.6	58.0
mTPS_10CA/PLA 50/50	53.1	104.4	15.8	152.7	14.1	30.3
mTPS_10CA/PLA 40/60	55.2	108.7	16.8	154.7	16.5	29.5
mTPS_20CA/PLA 60/40	55.4	108.4	14.60	153.2	13.4	36.0
mTPS_20CA/PLA 50/50	56.0	110.0	18.04	153.9	17.0	36.5
mTPS_20CA/PLA 40/60	56.0	109.7	20.15	155.0	19.4	34.7

*T_g_*—glass transition temperature; *T_c_*—crystallization temperature; *T_m_*—melting temperature; Δ*H_c_*—crystallization enthalpy; ∆*H_m_*—melting enthalpy; *χ_c_*—degree of crystallinity.

**Table 5:** Results of TGA analysis.

Sample	*T*_onset_ (°C)	*T*_max1_ (°C)	*T*_max2_ (°C)	*T*_end_ (°C)	*R*_600°C_ (%)
PLA	287.4	/	345.0	363.4	1.0
TPS	253.4	287.3	/	334.5	7.8
TPS/PLA 60/40	235.3	252.3	293.2	375.3	2.5
TPS/PLA 50/50	259.3	293.7	333.8	394.6	4.3
TPS/PLA 40/60	276.5	310.8	359.6	396.4	5.1
mTPS_5CA/PLA 60/40	258.7	295.3	354.5	393.6	3.4
mTPS_5CA /PLA 50/50	229.7	248.3	297.8	371.4	5.1
mTPS_5CA/PLA 40/60	232.9	308.7	319.2	391.4	5.2
mTPS_10CA /PLA 60/40	238.4	/	312.8	337.4	5.4
mTPS_10CA /PLA 50/50	225.7	257.3	288.6	363.4	4.8
mTPS_10CA /PLA 40/60	234.6	312.4	/	390.2	6.3
mTPS_20CA /PLA 60/40	222.4	285.4	294.8	324.5	7.3
mTPS_20CA /PLA 50/50	219.4	228.9	255.6	364.4	6.3
mTPS_20CA /PLA 40/60	218.4	258.4	276.4	342.5	10.4

*T_onset_*—initial degradation temperature; *T_max_*—temperature at maximum degradation rate; *T_end_*—final degradation temperature; *R_600°C_*—residue at 600 °C.

## Data Availability

The original contributions presented in this study are included in the article. Further inquiries can be directed to the corresponding author.
